# Antiretroviral therapy abrogates association between arginase activity and HIV disease severity^☆^

**DOI:** 10.1016/j.trstmh.2010.08.004

**Published:** 2010-11

**Authors:** T.E. Cloke, T. Abebe, A. Hailu, M. Munder, G.P. Taylor, I. Müller, P. Kropf

**Affiliations:** a Department of Immunology, Faculty of Medicine, Imperial College London, Norfolk Place, London W2 1PG, UK; b Department of Microbiology, Parasitology and Immunology, University of Addis Ababa, Addis Ababa, Ethiopia; c Department of Hematology and Oncology, University of Mainz, Mainz, Germany; d Department of Genito-Urinary Medicine and Communicable Diseases, Imperial College London, London, UK

**Keywords:** HIV, Immune response, T cells, CD4 cell count, Arginase, L-arginine

## Abstract

Arginase-induced L-arginine deprivation is emerging as a key mechanism for the downregulation of immune responses. We hypothesised that arginase activity increases with disease severity in HIV-seropositive patients. Our results show that peripheral blood mononuclear cells (PBMCs) from 23 HIV-seropositive patients with low CD4^+^ T cell counts (≤350 cells/μl) expressed significantly more arginase compared with 21 patients with high CD4^+^ T cell counts. Furthermore, we found a significant association between the two principal prognostic markers used to monitor HIV disease (CD4^+^ T cell count and plasma viral load) and PBMC arginase activity in antiretroviral therapy naïve patients but not in patients undergoing therapy.

## 1 Introduction

HIV results in a chronic infection that progressively impairs the immune system. Although depletion of CD4^+^ T cells explains much of the immunosuppression, the precise mechanisms involved in the onset of immunopathology during HIV infection have not yet been resolved.^1^

The metabolism of L-arginine by arginase is emerging as a crucial mechanism for the regulation of immune responses. L-arginine has two principal metabolic fates; either it can be metabolised by nitric oxide synthase into nitric oxide or by arginase into ornithine and urea. Arginase has been shown to impair T cell responses by reducing the bioavailability of L-arginine: high arginase activity expressed by myeloid cells results in reduced availability of extracellular L-arginine in the microenvironment. In turn, this decrease in L-arginine results in T cell hyporesponsiveness.^2^, ^3^, ^4^

To test the hypothesis that arginase activity is increased in HIV-seropositive (HIV+) patients and might contribute to immune dysfunction and disease progression we measured the levels of arginase activity in peripheral blood mononuclear cells (PBMCs) isolated from the blood of HIV+ patients.

## 2 Materials and methods

The duration of the study was from February 2008 to December 2009 and a cohort of 44 HIV+ patients was recruited from St Mary's Hospital, London UK. Inclusion criteria were (i) HIV+ by standard laboratory tests and (ii) older than 18 years. From the patient's hospital records it was determined whether the patient was receiving antiretroviral therapy (ART). All subjects gave written, informed consent before participation.

Plasma HIV-I viral RNA was quantified (Bayer Quantiplex assay; Bayer Diagnostics, East Walpole, MA, USA). The standard T lymphocyte markers, CD3, CD4 and CD8 were determined by flow cytometry.

Twenty millilitres of anticoagulated peripheral blood was collected in EDTA tubes and PBMCs were isolated by density gradient centrifugation on Histopaque^®^-1077 (Sigma Chemical Co., St. Louis, MO, USA).

The enzymatic activity of arginase was measured by a colorimetric enzymatic assay as previously described.^5^ Arginase activity is expressed as mU per ml of blood.

Data were evaluated for statistical differences using a two-tailed Mann-Whitney *U* test and for correlation using Spearman's rank test with GraphPad PRISM version 5.0 (Prism, San Diego, CA, USA).

## 3 Results

**Figure 1:**
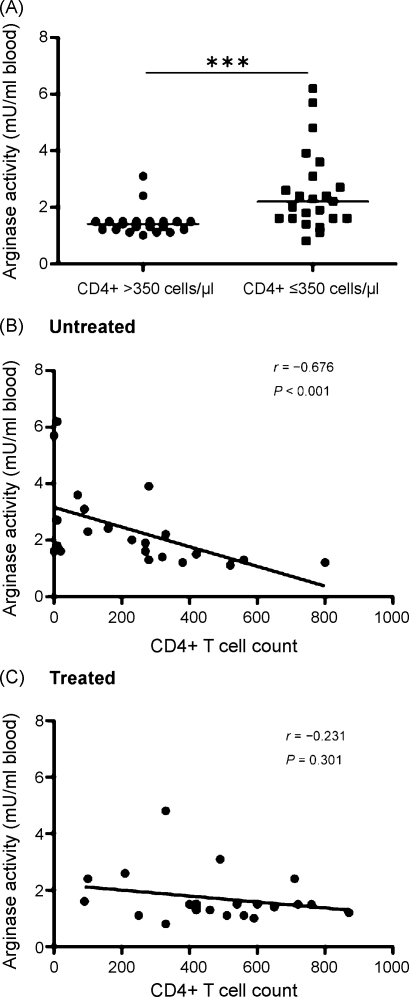
Arginase activity in peripheral blood mononuclear cells isolated from 44 HIV-seropositive patients. (A) Arginase activity in 21 patients with CD4^+^ T cell count > 350 cells/μl and 23 patients with CD4^+^ T cell count ≤350 cells/μl; ***P < 0.001. (B) Arginase activity and CD4^+^ T cell counts for 22 patients not on antiretroviral therapy (ART). (C) Arginase activity and CD4^+^ T cell counts for 22 patients on ART.

We subdivided our cohort of HIV+ patients into two groups based on their CD4^+^ T cell count. Arginase activity in PBMCs isolated from 23 HIV+ patients with low CD4^+^ T cell counts (≤350 cells/μl) was significantly higher than that in 21 HIV+ patients with high CD4^+^ T cell counts (median ± SEM: 2.2 ± 0.3 vs. 1.4 ± 0.1 mU/ml blood, respectively, *P* < 0.001; Figure 1A). Moreover, we found a statistically significant inverse correlation between arginase activity and CD4^+^ T cell count (*r* = −0.59, *P* < 0.001). In addition, our results show that high viral load correlates with high arginase activity (*r* = 0.43, *P* = 0.003).

To assess the impact of ART on arginase activity we stratified the cohort into two groups. The 22 patients on ART had a median (range) CD4^+^ T cell count of 475 (90–870) and 21 of them had an undetectable plasma viral load (<1.7 log10 copies/ml). The 22 patients not on ART had a median (range) CD4^+^ T cell count of 250 (0–800) and a median (range) plasma viral load of 5.1 (2.66-5.67) log10 copies/ml.

Interestingly, a highly significant inverse correlation was found between CD4^+^ T cell count and PBMC arginase activity in untreated but not in treated patients (untreated: *r* = −0.676, *P* < 0.001 vs. treated: *r* = −0.231, *P* = 0.301; Figures 1B and C). In addition, a positive association between plasma viral load and PBMC arginase activity was found in untreated patients (*r* = 0.47, *P* = 0.03). As 21 of the 22 patients receiving ART had viral loads below detection limits association between arginase activity and viral load in these patients could not be calculated.

These results show that both low CD4^+^ T cell count and high viral load correlate with high arginase activity in untreated but not treated HIV+ patients.

## 4 Discussion

Our study reveals that arginase activity is significantly higher in PBMCs from HIV+ patients with a low CD4^+^ T cell count, compared with that in HIV+ patients with a high CD4^+^ T cell count. Moreover, we found that in ART naïve patients there is a significant association between high PBMC arginase activity and both of the principal markers of HIV disease progression, namely low CD4^+^ T cell count and high plasma viral load. Therefore, we propose that the higher arginase activity detected in PBMCs from advanced untreated HIV+ patients may result in lower levels of L-arginine, thereby causing dysregulation of T cell responses. One potential consequence of L-arginine starvation is altered T cell proliferation as it has been shown that sub-physiological levels of L-arginine lead to G0-G1 cell cycle arrest.^4^ A better understanding of the events resulting in the upregulation of arginase and the subsequent downregulation of T cell responsiveness will improve our understanding of the complex mechanisms leading to progressive immune dysfunction in HIV/AIDS and may provide new targets to improve the efficacy of therapy.

## Authors’ contributions

All authors contributed to the conception and design of the study, selection of patients, laboratory analysis, data analysis and interpretation, and drafting of the manuscript. All authors contributed to and read and approved the final manuscript. IM and PK are guarantors of the paper.

## Funding

This work was supported by a grant from The Wellcome Trust (07664/Z/05/Z, PK) and TC is a recipient of an Imperial College London MB/PhD fellowship.

## Conflicts of interest

None declared.

## Ethical approval

The study protocol was approved by the UK NHS National Research Ethics Service (COREC reference 05/Q0410/93).
